# Mechanism of *Pasteurella multocida* Lysis by Virulent Phage vB_PmuP_Pa7: Insights from a Strand-Specific Transcriptome Analysis

**DOI:** 10.3390/microorganisms14071582

**Published:** 2026-07-20

**Authors:** Hongjian Zhang, Jinlin Ma, Wei Zhang, Wenliang Li, Jing Zhao, Xin Wei, Chunhui Feng, Yaqiong Fang, Fei Yang

**Affiliations:** 1 College of Agriculture and Animal Husbandry, Qinghai University, Xining 810016, China; 2 Hainbei Tibetan Autonomous Prefecture Animal Disease Prevention and Control Center, Haibei 810299, China; 3 College of Veterinary Medicine, Nanjing Agricultural University, Nanjing 210095, China; 4 Institute of Veterinary Medicine, Jiangsu Academy of Agricultural Sciences, Nanjing 210014, China; 5 Key Laboratory of Veterinary Biological Engineering and Technology, Ministry of Agriculture, Nanjing 210014, China

**Keywords:** *Pasteurella multocida*, virulent bacteriophage, Pa7, strand-specific transcriptome, transcriptomics, host–phage interaction, yak

## Abstract

Virulent phage vB_PmuP_Pa7 is a promising candidate for controlling *Pasteurella multocida* infection, yet the molecular basis of its lytic process remains poorly understood. Here, we performed strand-specific RNA sequencing on *P. multocida* S34 infected with Pa7 at 0, 20, 30, and 120 min to characterize phage–host transcriptional dynamics. A total of 234, 433, and 558 differentially expressed genes (DEGs) were detected at 20, 30, and 120 min, respectively, indicating progressive host reprogramming during infection. PCA, expression distribution analysis, and sample correlation analysis confirmed clear stage-specific transcriptional shifts. GO and KEGG enrichment analyses indicated that Pa7 infection was associated with changes in translation, ribosome function, ABC transporters, amino sugar and nucleotide sugar metabolism, bacterial chemotaxis, and the TCA cycle. Four representative differentially expressed genes involved in transport, carbohydrate metabolism, and translation-related functions were selected for RT-qPCR analysis. The RT-qPCR results showed partial, gene- and time-point-dependent agreement with the RNA-seq-derived expression patterns. Together, these findings provide a time-resolved transcriptional profile of Pa7 infection and identify host pathways potentially associated with the infection process and bacterial cell lysis. However, the direct functional contributions of these genes and pathways remain to be experimentally determined.

## 1. Introduction

*Pasteurella multocida* is an important animal pathogen with a broad host range. It can cause respiratory disease, septicemia, and secondary infections in cattle, yaks, pigs, and poultry, leading to persistent economic losses in livestock production worldwide. In high-altitude pastoral areas, this bacterium is closely linked to hemorrhagic septicemia in yaks and bovine respiratory disease, making it a major target for regional animal-health control. Antibiotics have long been the main strategy for controlling *P. multocida* infection; however, prolonged, high-dose, and inappropriate antimicrobial use in clinical and farm settings has accelerated the emergence and spread of resistant strains, thereby reducing treatment efficacy and increasing the difficulty of disease control [1,2,3,4]. Antimicrobial resistance in *P. multocida* and other Gram-negative bacterial pathogens may be associated with multiple mechanisms, including β-lactamase production, reduced membrane permeability, efflux pump activity, and target-site modification. These mechanisms can reduce antimicrobial efficacy and complicate the control of bacterial respiratory infections in livestock.

Against the backdrop of intensifying antimicrobial resistance, bacteriophages—natural viruses that specifically recognize and lyse bacteria—have regained widespread attention. Compared with antibiotics, lytic phages offer high host specificity, self-amplification at the infection site, limited disturbance to the commensal microbiota, and the potential for synergy with antibiotics [5,6,7,8,9,10]. In recent years, phage cocktails, phage–antibiotic combinations, and phage engineering have steadily advanced, moving phage therapy from proof of concept toward more realistic translational use [11,12,13,14,15,16,17]. For animal pathogens such as *P. multocida*, phages may not only serve as alternative antibacterial agents but may also contribute to vaccine development, companion diagnostics, and biocontrol strategies [18,19,20].

In recent years, increasing attention has been paid to bacteriophages targeting *P. multocida* and other respiratory bacterial pathogens of livestock and poultry [18,19,21,22]. Several virulent or lytic phages infecting *P. multocida* have been isolated and characterized in terms of morphology, biological properties, host range, adsorption capacity, one-step growth characteristics, genomic features, and bactericidal activity [18,19,22]. These studies suggest that *P. multocida* phages can reduce bacterial loads and may serve not only as promising antibacterial candidates for the control of pasteurellosis but also as useful tools for understanding bacterial surface structures, host specificity, and pathogen adaptation [18,23]. Moreover, phage cocktails and phage–antibiotic combinations have been explored to broaden the antibacterial spectrum and reduce the emergence of resistant bacterial variants [24]. However, most previous studies have mainly focused on phage isolation and phenotypic or genomic characterization, whereas the molecular responses of *P. multocida* to phage infection remain poorly understood, particularly at the transcriptomic level. Therefore, time-resolved, strand-specific RNA-seq provides an opportunity to characterize host transcriptional reprogramming at different stages of the lytic cycle and to identify pathways potentially associated with phage-induced bacterial lysis [25,26].

High-throughput RNA sequencing has provided a new systems-biology perspective for dissecting phage–host interactions. Previous studies have shown that lytic phage infection rapidly reprograms the host transcriptome and that phage gene expression often follows an early–middle–late temporal pattern; meanwhile, major changes are also observed in translation, energy metabolism, membrane transport, and defense systems of the host cell [25,27,28]. Transcriptomic studies in *Staphylococcus aureus*, *Pseudomonas aeruginosa*, *Bacteroides*, and other model systems have confirmed that phage infection can profoundly affect ribosomes, ABC transport systems, stress responses, and metabolic flux allocation [29,30,31,32,33,34]. Our previous characterization of the yak-derived virulent phage vB_PmuP_Pa7 and its host strain *P. multocida* S34 showed that Pa7 has a short latent period, a clear rise period, and strong lytic activity against capsular serotype B strains. These data provided the basis for selecting the infection time points used in the present transcriptomic analysis. In the present study, we used strand-specific transcriptome sequencing to systematically characterize temporal transcriptomic changes during Pa7 infection at 0, 20, 30, and 120 min. GO/KEGG enrichment and RT-qPCR validation were then applied to clarify the molecular mechanism by which Pa7 lyses *P. multocida*, with the goal of providing a theoretical basis for phage-based alternatives to antibiotics.

## 2. Materials and Methods

### 2.1. Bacterial Strain, Phage, and Culture Conditions

*P. multocida* strain S34, a capsular serotype B strain isolated from yak, was a capsular serotype B strain that was isolated and preserved in our laboratory; its corresponding lytic phage, vB_PmuP_Pa7, was isolated and preserved by the Laboratory of Infectious Diseases, Qinghai University. According to our previous characterization of its biological properties, vB_PmuP_Pa7 has a relatively narrow host range and exhibits lytic activity only against capsular serotype B strains of *P. multocida*, whereas no obvious lytic activity was observed against serotype A, D, E, or F strains. In this study, the host strain S34 was grown in brain heart infusion (BHI) broth at 37 °C with shaking at 180 rpm to the mid-logarithmic phase and then used for subsequent infection assays.

### 2.2. Phage Infection and Sample Collection

**Figure 1:**
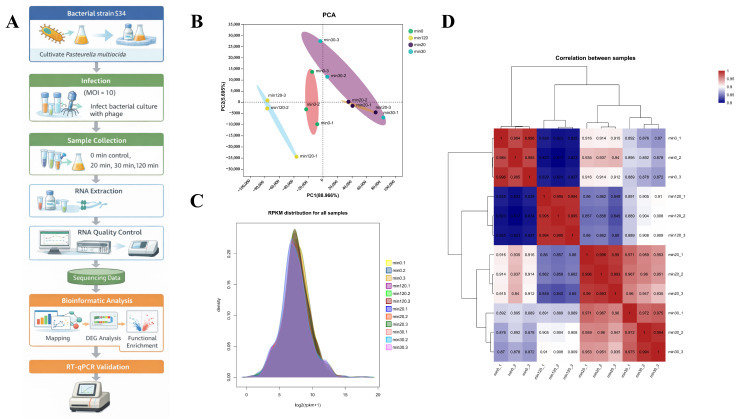
Experimental design and global transcriptomic analysis of Pa7 infection in *P. multocida* S34: (**A**) Schematic of the experimental workflow and sampling time points. (**B**) PCA plot showing sample separation across infection stages; the percentages on the axes indicate the variance explained by PC1 and PC2. (**C**) Gene expression distribution across samples. (**D**) Heatmap of pairwise sample correlations.

To accurately capture the temporal transcriptional responses of the host during phage infection, the transcriptomic sampling time points in this study were determined based on the one-step growth curve of phage vB_PmuP_Pa7 obtained in our previous work. Specifically, the latent period was approximately 15–20 min, the rise period extended from 20 to 120 min, and a plateau phase was reached after 120 min, with an average burst size of 162 PFU/cell. On this basis, and with reference to the transcriptomic sampling strategy reported by Zhang et al. [35], an infection model was established under a high multiplicity of infection (MOI = 10). Briefly, the host culture was grown to approximately 1 × 10^9^ CFU/mL (OD600 ≈ 0.6), followed by infection with phage vB_PmuP_Pa7. Samples were collected at 0 min before infection (uninfected control), 20 min (end of the latent period), 30 min (early rise period), and 120 min (late rise/initial plateau stage) (Figure 1A). At each time point, bacterial cultures were rapidly collected in three biological replicates, immediately mixed with RNA stabilization reagent or processed for RNA extraction, snap-frozen in liquid nitrogen, and stored at −80 °C until RNA isolation/extraction; all samples were processed using the same procedure to minimize RNA degradation and technical variation.

### 2.3. Total RNA Extraction and Quality Control

Total RNA was extracted from the samples using TRIzol^®^ Reagent (Invitrogen, Carlsbad, CA, USA) according to the manufacturer’s instructions, and residual genomic DNA was removed using DNase I (Takara Bio Inc., Shiga, Japan). RNA concentration and purity were assessed using a NanoDrop ND-2000 spectrophotometer (Thermo Fisher Scientific, Wilmington, DE, USA), and RNA integrity was evaluated using an Agilent 2100 Bioanalyzer (Agilent Technologies, Santa Clara, CA, USA) based on electropherogram quality, RIN values, and the integrity of bacterial rRNA peaks. The eukaryotic 28S/18S rRNA ratio was not used for bacterial RNA quality assessment.

### 2.4. Library Preparation and Illumina HiSeq Sequencing

Strand-specific RNA-seq libraries were prepared using a ribosomal RNA-depleted bacterial RNA library preparation workflow. Briefly, rRNA was removed from total RNA using the Ribo-Zero rRNA Removal Kit (Illumina, San Diego, CA, USA). The remaining RNA was fragmented, followed by first-strand and second-strand cDNA synthesis, end repair, A-tailing, adapter ligation, and PCR amplification according to the manufacturer’s instructions. Libraries with insert sizes of approximately 200–300 bp were selected and quantified before sequencing. Paired-end sequencing was performed by Shanghai BIOZERON Co., Ltd. (Shanghai, China) on an Illumina NovaSeq 6000 platform (Illumina, San Diego, CA, USA), with a read length of 150 bp × 2. Because bacterial transcripts generally lack poly(A) tails, the library preparation workflow was based on rRNA depletion rather than poly(A) selection, making it suitable for bacterial strand-specific RNA-seq.

### 2.5. Read Quality Control and Mapping

Raw paired-end reads were first assessed using FastQC (version 0.12.1), and adapter sequences and low-quality bases were removed using Trimmomatic version 0.36 (https://github.com/usadellab/Trimmomatic, accessed on 29 April 2023) with the parameters SLIDINGWINDOW:4:15 and MINLEN:75. Clean reads were retained for downstream analysis only after quality filtering. The quality of sequencing data was evaluated based on Q20, Q30, GC content, error rate, and the consistency among biological replicates. Clean reads were mapped to the reference genome of *P. multocida* S34 and the genome of phage vB_PmuP_Pa7 using Rockhopper (https://cs.wellesley.edu/~btjaden/Rockhopper/, accessed on 29 April 2023) in strand-specific mode. The corresponding genome annotation files in GFF/GTF format were used for gene-level read assignment. To further evaluate the infection-associated changes in read origin, reads were also aligned to a combined host–phage reference genome. Mapping statistics for host-derived and phage-derived reads were summarized for each sample.

Strand-specific RNA-seq data were generated from *P. multocida* S34 samples collected at 0, 20, 30, and 120 min after infection with phage vB_PmuP_Pa7.

### 2.6. Differential Expression Analysis and Functional Enrichment

Gene-level raw read counts generated from the mapping results were used as input for edgeR (https://bioconductor.org/packages/release/bioc/html/edgeR.html, accessed on 29 April 2023). Raw counts were normalized using the trimmed mean of M-values (TMM) method to account for differences in library size and RNA composition among samples. Dispersion was estimated using the edgeR (https://bioconductor.org/packages/release/bioc/html/edgeR.html, accessed on 29 April 2023) pipeline, and differential expression analysis was performed based on the negative binomial model. *p*-values were adjusted for multiple testing using the Benjamini–Hochberg false discovery rate (FDR) method. Genes with |log_2_FC| > 1 and FDR < 0.05 were considered differentially expressed genes (DEGs). RPKM values were used only for descriptive visualization of gene expression profiles and were not used as input for differential expression analysis. GO enrichment and KEGG pathway enrichment analyses were performed using Goatools (https://github.com/tanghaibao/Goatools, accessed on 29 April 2023) and KOBAS (https://github.com/xmao/kobas, accessed on 29 April 2023), respectively. Enriched GO terms and KEGG pathways with adjusted *p*-values < 0.05 were considered statistically significant.

### 2.7. Validation by RT-qPCR

To examine selected expression patterns identified by RNA-seq, four DEGs, namely DR93_RS03530, DR93_RS01060, DR93_RS04575, and DR93_RS01265, were selected based on their differential-expression profiles and their representation of transport, carbohydrate metabolism, and translation-related functions for RT-qPCR analysis. Gene-specific primers were designed using Geneious Pro 4.8.5 (Table 1), and the KMT1 gene was used as the internal reference. For each primer pair, amplification efficiency, standard-curve slope, R^2^ value, melting-curve specificity, and Ct range were evaluated. Only primers showing a single melting peak and acceptable amplification efficiency were used for subsequent analysis. qPCR was performed using Hieff^®^ qPCR SYBR Green Master Mix (Yeasen Biotechnology (Shanghai) Co., Ltd., Shanghai, China) on a QuantStudio 3 Real-Time PCR System (Thermo Fisher Scientific, Waltham, MA, USA). The thermal cycling conditions were as follows: initial denaturation at 95 °C for 30 s, followed by 35 cycles of 95 °C for 5 s and 60 °C for 34 s. Each sample was run in triplicate. The relative expression levels of the target genes were calculated using the 2^(−ΔΔCt) method.

### 2.8. Statistical Analysis

RNA-seq differential expression analysis was performed using edgeR as described above. For RT-qPCR and other non-RNA-seq quantitative data, values are presented as the mean ± standard deviation (SD) from three biological replicates. Normality and homogeneity of variance were assessed where applicable. Differences between the two groups were evaluated using Student’s *t*-test, whereas comparisons among multiple groups were evaluated using one-way analysis of variance (ANOVA). *p*-values < 0.05 were considered statistically significant. Agreement between RNA-seq and RT-qPCR was evaluated by comparing the direction and temporal pattern of expression changes for each selected gene. Because only four genes were examined, the RT-qPCR results were interpreted as targeted supporting evidence for selected transcriptional changes rather than as comprehensive validation of the entire transcriptomic dataset.

## 3. Results

### 3.1. Phage vB_PmuP_Pa7 Manipulates the Transcriptional System of Host Bacterium S34

Transcriptome sequencing was performed on 12 samples from four time points (0, 20, 30, and 120 min). The raw reads obtained for each sample ranged from 22.76 to 36.00 million. After stringent quality control, the Q20 and Q30 scores for all samples were above 98.04% and 93.75%, respectively; the error rates were below 0.025%; and the GC content was stable between 42.95% and 45.19% (Table S1), indicating exceptionally high sequencing quality. The clean reads were then aligned to the reference genome, with mapping rates ranging from 34.93% to 63.41% (Table S2). Because the initial mapping was performed mainly against the host genome, the reduced host mapping rate at later infection stages may be explained by the increasing abundance of phage-derived transcripts, host RNA degradation, and progressive cell lysis. Therefore, reads were further evaluated using a combined host–phage reference to better distinguish host-derived and phage-derived transcript populations. Notably, the mapping rate decreased significantly at the late infection stage (120 min), suggesting a reduced proportion of host-derived transcripts, potentially due to extensive phage replication and/or host RNA degradation.

The reduced host-genome mapping rate observed at the late infection stage should be interpreted cautiously. Because the original alignment was primarily performed against the host reference genome, the decrease in host-mapped reads at 120 min may reflect an increased proportion of phage-derived transcripts, degradation of host RNA, progressive host-cell disruption, and/or incomplete representation of infection-derived transcripts in the host-only reference. When reads were evaluated against the combined host–phage reference, the relative contribution of phage-derived reads increased during infection, supporting the interpretation that Pa7 infection progressively shifts the transcript population from host-dominated to phage-associated RNA. Therefore, the decline in host mapping rate is not simply a technical artifact but may also reflect the biological progression of Pa7 replication and host-cell lysis.

### 3.2. Global Analysis of Gene Expression Patterns During Infection

To assess the relationships between samples, we performed principal component analysis (PCA). The results showed that the control (0 min) and the late rise period (120 min) samples separated distinctly, while the latent period (20 min) and early rise (30 min) groups clustered closely along the first principal component (Figure 1B), indicating a phase-specific transcriptional reprogramming of the host in response to phage infection. The close clustering of the 20 min and 30 min samples suggests transcriptional continuity between the end of the latent period and the early rise period. However, the increased number of DEGs and the stronger enrichment of metabolic and translational pathways at 30 min indicate that this time point may represent a transition from early host response to more pronounced transcriptional reprogramming. In contrast, the clear separation of the 120 min samples from the earlier stages indicates substantial remodeling of the host transcriptome during the late rise/initial plateau stage, which is consistent with extensive phage replication and progressive host-cell disruption. The gene expression probability density distribution further confirmed this, with nearly identical curves for 0 min and 20 min, and similar peak patterns between 30 min and 120 min that were distinct from the early stages (Figure 1C), suggesting the emergence of a highly expressed gene population in the late phase of infection. Sample correlation analysis demonstrated high reproducibility among biological replicates and lower correlations between early and late infection samples, reinforcing the phased transcriptional changes (Figure 1D).

### 3.3. Temporal Dynamics of Differentially Expressed Genes Induced by Phage Infection

**Figure 2:**
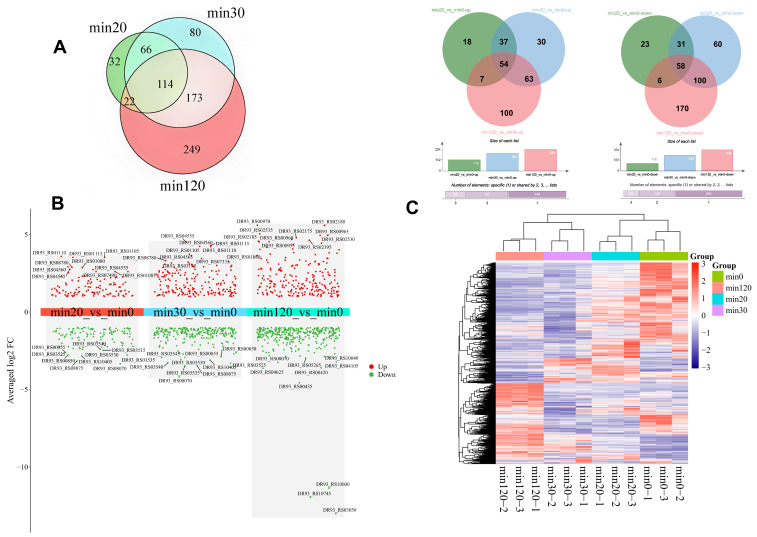
Temporal dynamics of DEGs during Pa7 infection. DEGs were identified using the threshold |log_2_FC| > 1 and FDR < 0.05: (**A**) Number of up- and downregulated DEGs at each infection stage. (**B**) Volcano plots showing DEGs in each comparison. (**C**) Hierarchical clustering heatmap of DEGs based on normalized expression values; clustering was performed using Euclidean distance and complete linkage.

Using thresholds of |log_2_FC| > 1 and FDR < 0.05, we systematically identified DEGs at each infection stage compared to the control (Table S3). The number of DEGs increased progressively throughout the infection: 234 (116 upregulated, 118 downregulated) at the latent period (20 min vs. 0 min), 433 (184 upregulated, 249 downregulated) at the early rise period (30 min vs. 0 min), and 558 (224 upregulated, 334 downregulated) at the late rise period (120 min vs. 0 min) (Figure 2A). Venn and differential-expression analyses revealed common and unique DEGs between the different comparison groups (Figure 2B). A total of 54 upregulated DEGs (Table 2) and 58 downregulated DEGs (Table 3) were identified. In addition, the samples were analyzed by heatmap clustering and divided into four clusters (Figure 2C), indicating that the host response to phage infection is a dynamic and complex process involving the temporal regulation of distinct gene modules.

### 3.4. Functional Enrichment Analysis of Differentially Expressed Genes

**Figure 3:**
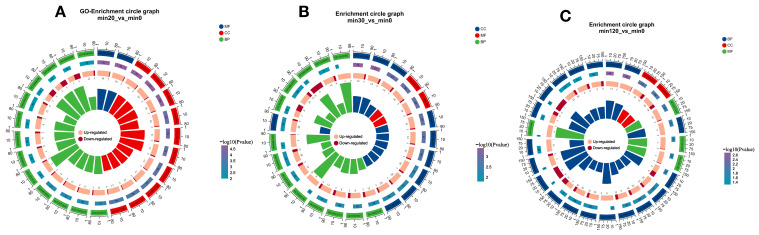
GO functional classification of Pa7-induced genes: (**A**–**C**) GO pathway enrichment results at 20, 30, and 120 min post-infection (relative to 0 min), respectively.

To elucidate the biological functions of the DEGs, GO and KEGG enrichment analyses were conducted. GO analysis revealed that DEGs were significantly enriched in Biological Process terms such as ‘translation’, ‘carbohydrate metabolic process’, and ‘transmembrane transport’. For Molecular Function, the most enriched terms were ‘ATP binding’, ‘structural constituent of ribosome’, and ‘DNA binding’ (Figure 3A–C). Finer analysis of individual comparisons showed that early-stage DEGs (min20 vs. min0) were primarily related to basic cellular processes and metabolism (Figure 3A), whereas mid- and late-stage DEGs were more involved in transcription regulatory activity and stress response (Figure 3B,C).

**Figure 4:**
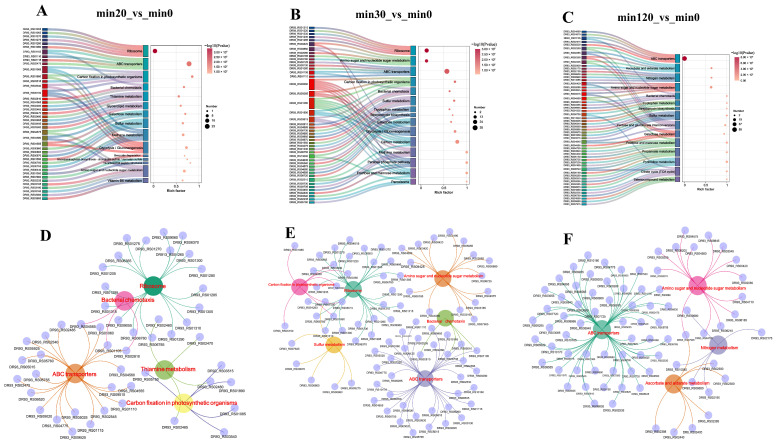
KEGG functional classification of Pa7-induced genes: (**A**–**C**) KEGG pathway enrichment results at 20, 30, and 120 min post-infection (relative to 0 min), respectively; (**D**–**F**) representative stage-associated enriched functional categories at 20, 30, and 120 min post-infection, respectively.

KEGG pathway analysis further revealed the systemic molecular mechanisms underlying phage infection. The core pathways commonly and significantly enriched across all DEGs included ribosomes, ABC transporters, amino sugar and nucleotide sugar metabolism, bacterial chemotaxis, and the TCA cycle (Figure 4A–C). The enrichment of ribosome- and translation-related terms suggests that Pa7 infection is associated with marked perturbation of the host protein synthesis machinery. Changes in ABC transporters and TRAP transporters likely reflect altered nutrient uptake, membrane-associated physiological remodeling, and stress adaptation rather than direct evidence of phage adsorption. Enrichment of amino sugar and nucleotide sugar metabolism may be related to changes in cell-envelope precursor metabolism, which is particularly relevant to *P. multocida* because surface structures such as the capsule and lipopolysaccharide are important for bacterial physiology and host interaction. Alterations in the TCA cycle further suggest that energy metabolism is affected during infection, potentially reflecting the increased metabolic burden imposed by phage replication and the progressive loss of host cellular homeostasis. In-depth analysis at different time points indicated that pathways related to nucleotide synthesis (e.g., purine and pyrimidine metabolism) and energy metabolism were significantly activated early in infection (Figure 4D). In contrast, pathways associated with cell wall/membrane biosynthesis and stress responses were more altered during the later stages (Figure 4E,F). These enrichment patterns indicate that Pa7 infection is associated with broad changes in host transcriptional and metabolic pathways, particularly those related to protein synthesis, nutrient transport, cell-envelope precursor metabolism, and energy metabolism. However, pathway enrichment analysis provides associative evidence and does not establish that Pa7 directly regulates these pathways or that the identified pathways are required for phage replication or bacterial lysis. Further functional experiments will be necessary to determine the specific roles of these pathways during Pa7 infection.

### 3.5. Identification and Experimental Validation of Key Functional Genes

Based on the differential-expression and functional-enrichment analyses, four DEGs representing transport, carbohydrate metabolism, and translation-related functions were selected for RT-qPCR analysis. DR93_RS03530 is annotated as a TRAP transporter permease, DR93_RS01060 as an ABC transporter substrate-binding protein, DR93_RS04575 as a galactose-1-phosphate uridylyl transferase, and DR93_RS01265 as the 30S ribosomal protein S17. These functional annotations suggest that the selected genes may be associated with changes in membrane transport, carbohydrate metabolism, and translation during Pa7 infection. However, the present data do not demonstrate that these genes directly participate in phage adsorption, genome replication, infection amplification, or host-cell lysis.

**Figure 5:**
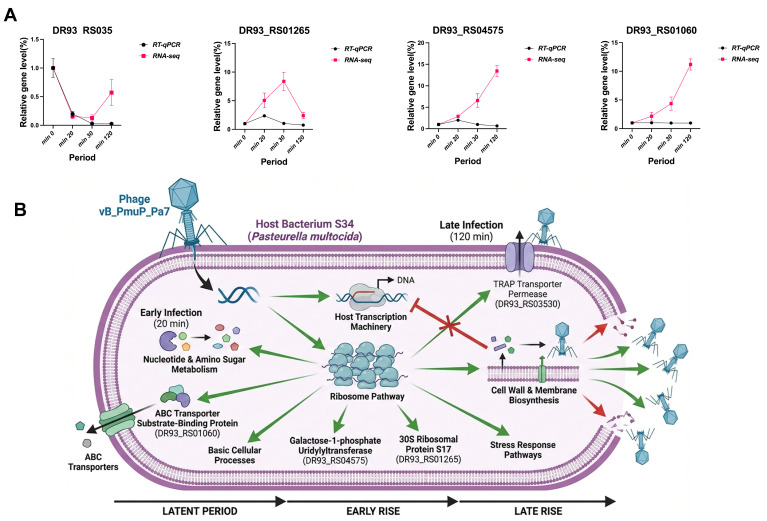
Comparison of RT-qPCR and RNA-seq expression patterns and a transcriptome-informed working model of host responses during Pa7 infection: (**A**) Temporal expression profiles of DR93_RS03530, DR93_RS01265, DR93_RS04575, and DR93_RS01060 determined by RT-qPCR and RNA-seq. Data are presented as the mean ± standard deviation from three biological replicates. For visualization and comparison, RT-qPCR relative-expression values were normalized to KMT1 and calculated using the 2^(−ΔΔCt) method with the 0 min uninfected control as the calibrator, whereas RNA-seq expression changes were plotted relative to the same control. Black lines and symbols represent RT-qPCR results, whereas magenta lines and symbols represent RNA-seq results. The panel is intended to compare the direction and temporal pattern of expression changes; the level of agreement varies among genes and time points. (**B**) Transcriptome-informed working model summarizing the major host pathways associated with Pa7 infection at 20, 30, and 120 min. Green arrows connect the infection-associated host processes included in the working model. Red arrows and crossed symbols indicate hypothesized inhibitory or disruptive events, black horizon-tal arrows indicate temporal progression through the infection process. The arrows and proposed relationships are inferred from differential-expression and enrichment analyses and do not by themselves demonstrate direct causal interactions.

The RT-qPCR and RNA-seq results showed similar directions or temporal patterns for some gene–time-point comparisons, whereas differences in expression magnitude and/or temporal pattern were observed for other comparisons, particularly at the later infection stage (Figure 5A). Therefore, the RT-qPCR results provide targeted support for selected RNA-seq-derived expression changes, but the level of agreement was not uniform across all genes and time points. These results should not be interpreted as comprehensive validation of all DEGs identified in the transcriptomic analysis.

### 3.6. Transcriptome-Informed Working Model of Host Responses During Pa7 Infection

Based on the strand-specific transcriptomic data, we constructed a transcriptome-informed working model to summarize the major host pathways altered during Pa7 infection (Figure 5B). Pa7 infection was associated with time-dependent changes in genes related to translation and ribosome function, transmembrane transport, amino sugar and nucleotide sugar metabolism, bacterial chemotaxis, and the TCA cycle. These transcriptional changes suggest progressive remodeling of host physiological processes during the infection cycle.

The four genes examined by RT-qPCR represent transporter-, carbohydrate-metabolism-, and ribosome-related functions. Because the agreement between RNA-seq and RT-qPCR varied among genes and time points, and because the present study did not include functional gene perturbation, protein-level validation, metabolite measurements, or direct assessment of pathway activity, the proposed model should be regarded as a hypothesis-generating summary rather than as a demonstrated causal mechanism of bacterial lysis. The direct contributions of individual genes and pathways to Pa7 replication and host-cell lysis remain to be established.

## 4. Discussion

This study used strand-specific transcriptomics to systematically reveal the stage-dependent transcriptional reprogramming of *P. multocida* S34 by the virulent phage vB_PmuP_Pa7. In this study, early, middle, and late stages refer to temporal phases of host transcriptome reprogramming during Pa7 infection rather than to canonical early, middle, and late phage gene-expression classes. As infection progressed from the latent phase to the early rise and late rise phases, the host transcriptome shifted markedly, and the number of differentially expressed genes increased continuously to 234, 433, and 558 at 20, 30, and 120 min, respectively, indicating that Pa7 did not merely perturb the host at a single time point; rather, it progressively commandeered host transcriptional resources throughout the lytic cycle. PCA and correlation analyses further showed that the 0 min and early-infection samples clustered closely, whereas the 120 min samples were clearly separated, suggesting that phage infection drove the host into distinct molecular states. This stage-specific pattern is highly consistent with the “early–middle–late” temporal programs described in other lytic phage systems and with the progressive replacement of host transcript dominance reported in previous transcriptomic studies [25,27,28,31].

GO and KEGG analyses showed that Pa7 infection primarily perturbed basic functional modules such as translation, ribosome structure, carbohydrate metabolism, transmembrane transport, ATP binding, and DNA binding. At the pathway level, ribosome, ABC transporters, amino sugar and nucleotide sugar metabolism, bacterial chemotaxis, and the TCA cycle were the most prominent shared enrichment terms. This indicates that Pa7-mediated lysis is not simply a destructive process; instead, the phage appears to have first remodeled the host protein synthesis and transport network to redistribute cellular resources in support of its own replication. In particular, enrichment of ribosome- and translation-related pathways suggests that Pa7 may rapidly gain control over the host protein synthesis machinery early in infection, whereas changes in amino sugar and nucleotide sugar metabolism, the TCA cycle, and energy-related pathways reflect deeper mobilization of precursors and energy in later stages. Similar metabolic hijacking has been reported for T4, phiR1-37, and other lytic phages, implying that phage infection often prioritizes reprogramming host resource allocation rather than simply inducing cell death [25,33,35].

ABC transport systems repeatedly emerged in this study, suggesting that they may represent a major entry point through which Pa7 reprograms host metabolism and membrane homeostasis. ABC transporters are not only responsible for nutrient uptake and ion balance but also participate in membrane potential maintenance, metabolic substrate import, and stress adaptation; therefore, altered expression usually reflects strong physiological remodeling in bacteria. At the same time, significant changes in bacterial chemotaxis-related pathways indicate that the host may alter motility, adhesion, and environmental sensing during infection, thereby influencing downstream responses after phage adsorption. Taken together with the changes in the TCA cycle and nucleotide metabolism, these findings suggest that Pa7 synchronously interferes with transmembrane transport, energy production, and signal response, compressing host survival capacity and shifting the cell toward a phage-replication mode. *P. multocida* itself already exhibits strong host adaptation and surface plasticity, and phage infection likely amplifies these changes, pushing the host into a rapidly destabilized state [4,36]. The four genes examined by RT-qPCR were selected to represent transporter-, carbohydrate-metabolism-, and ribosome-related functions identified in the transcriptomic analysis. Changes in DR93_RS03530 and DR93_RS01060 may reflect alterations in membrane transport, nutrient acquisition, or stress-associated physiological processes. Similarly, changes in DR93_RS04575 and DR93_RS01265 suggest that carbohydrate metabolism and ribosome-related functions were affected during infection. However, the present transcriptomic data do not establish that these genes function as phage receptors or directly promote adsorption, replication, infection efficiency, or host-cell lysis [30,31,32,35]. RT-qPCR reproduced the direction or temporal pattern of some RNA-seq-derived expression changes, whereas other gene–time-point comparisons differed in magnitude or temporal pattern. These differences may be related to the distinct normalization procedures, dynamic ranges, and measurement characteristics of the two methods. Accordingly, the RT-qPCR data should be interpreted as targeted support for selected transcriptional changes rather than as evidence of uniform agreement or comprehensive validation of the complete DEG dataset.

The TRAP permease and ABC substrate-binding protein suggest that Pa7 may enhance infection efficiency by regulating substrate transport across the membrane. The galactose-1-phosphate uridylyltransferase points to carbohydrate metabolism and the synthesis of cell wall/membrane precursors as important nodes that are likely prioritized during infection. The change in 30S ribosomal protein S17 further reflects phage-driven reorganization of the host translation apparatus. The strong concordance between RT-qPCR and RNA-seq indicates that these changes are true transcriptional responses rather than statistical noise during the Pa7 infection cycle. Consistently, other phage transcriptomic studies have also identified transport proteins, ribosomal proteins, and carbohydrate metabolism-related genes as key nodes in host takeover [30,31,32,35]. A limitation of this study is that RT-qPCR validation was performed for only four representative DEGs. Although these genes were selected based on expression magnitude, functional relevance, and pathway representation, additional genes and protein-level assays should be included in future studies to further validate the transcriptomic findings.

From a broader pathogenetic perspective, the virulence and host adaptation of *P. multocida* have long been linked to the capsule, LPS, iron acquisition systems, outer membrane proteins, and metabolic reprogramming [1,4]. Therefore, the transcriptional regulation pattern observed here is not surprising; rather, it further confirms that this bacterium rapidly adjusts its metabolic and stress-response networks under phage pressure. Meanwhile, research on Pasteurella phages has expanded steadily in recent years: PHB01, CFP3, and other lytic phages have all shown lytic potential against *P. multocida* and were considered promising for therapy, vaccine-associated development, or biocontrol [18,19,20]. Collectively, these studies indicate that *P. multocida* phages are worth not only isolating and characterizing but also investigating through transcriptomic, proteomic, and in vivo validation to clarify their molecular mechanisms. The present work is a meaningful step in that direction.

From the perspective of host defense, bacteria and phages are engaged in an ongoing arms race. Restriction–modification systems, CRISPR-Cas systems, toxin–antitoxin systems, and several newly identified defense islands can all limit phage replication, whereas phages counter through DNA modification, receptor changes, anti-CRISPR mechanisms, and regulation of host RNA stability [28]. Although this study did not directly dissect the receptor or lytic enzymes of Pa7, the host transcriptome clearly shows that Pa7 successfully overcame basal host defenses and efficiently reprogrammed the cell, indicating strong infectious adaptability. For veterinary applications, mechanistic clarification of Pa7 has two important implications: first, it supports phage substitution as a feasible strategy for controlling *P. multocida*; second, it provides a basis for more complex application schemes such as phage cocktails, phage–antibiotic combinations, and phage-based marker vaccine development [6,8,10,11,15,17].

Although this study provides a relatively complete transcriptomic portrait of Pa7 infection in *P. multocida*, several limitations remain. First, the conclusions are based primarily on RNA-level evidence, and cross-validation with proteomics, metabolomics, and functional knockout experiments is still lacking; second, the current study used a high-MOI synchronous infection model, which may be better suited to revealing an “idealized” infection process, whereas interpretation under natural low-MOI, asynchronous, and host-microenvironment conditions should be made cautiously; third, the receptor recognition, DNA injection, replication control, and lysis execution modules of Pa7 have not yet been systematically dissected. Future studies should integrate time-resolved proteomics, metabolomics, gene editing, and animal infection models to validate how Pa7 reshapes host metabolism and defense networks and to evaluate its practical application in yaks or other ruminants. Even so, the present work suggests that Pa7 achieves efficient lysis by reprogramming host transcription and metabolic networks, thereby laying a solid molecular foundation for phage-based strategies against *P. multocida* [37].

The present study provides a time-resolved transcriptomic profile of *P. multocida* S34 during Pa7 infection and identifies host pathways potentially associated with translation, transport, energy metabolism, and cell-envelope precursor metabolism. However, the study is limited by the lack of proteomic validation, functional gene assays, and in vivo evaluation. Therefore, future studies should combine transcriptomics with proteomics, metabolomics, and targeted genetic experiments to verify the functional roles of the candidate genes and pathways identified here.

## 5. Conclusions

This study characterized time-resolved transcriptional changes in *P. multocida* S34 during infection with the virulent phage vB_PmuP_Pa7. Pa7 infection was associated with changes in host pathways related to translation, ribosome function, transport, carbohydrate metabolism, cell-envelope precursor metabolism, and energy metabolism. RT-qPCR analysis of four selected genes provided partial support for the RNA-seq-derived expression patterns, with the level of agreement varying among genes and time points. These results identify candidate host genes and pathways for future functional investigation but do not by themselves establish that the identified pathways causally mediate phage replication or bacterial lysis. The present study provides a transcriptomic framework for subsequent mechanistic and application-oriented investigations of Pa7 infection.

## Figures and Tables

**Table 1:** Primers for RT-qPCR.

Gene	Primers (5′-3′)	Product Length (bp)
RS03530	F: ATGTCACTCACCAGCACTT R: GTATCATTAACCCTTCCAC	102
RS01060	F: TGGGTTGATTCTTGGTTAG R: AACGACTGCGTCAATGTTA	87
RS04575	F: AACCAACCGAACACCCACA R: TTTCATCCTCAGCCACTTT	120
RS01265	F: GCTTCGTTGTTCTCGTCATGC R: TCGTAGCGTGCAAGGTAAAGT	144
KMT1	F: TGCTCGTTGTGAGTGGGCT R: CGCTCTGTCGTTAATGGCTTC	253

**Table 2:** DEGs that were upregulated in each infection stage compared with the control group.

Gene ID	min20_vs._min0		min30_vs._min0		min120_vs._min0	
	logFC	*p*-Value	logFC	*p*-Value	logFC	*p*-Value
DR93_RS00530	1.163614246	8.89 × 10^−6^	1.294505071	7.47 × 10^−10^	2.335932095	7.57 × 10^−20^
DR93_RS00545	1.077074981	4.10 × 10^−5^	1.680815303	1.13 × 10^−13^	2.038879614	4.32 × 10^−16^
DR93_RS00550	1.404650392	1.45 × 10^−7^	1.793579204	6.46 × 10^−15^	2.326189738	2.49 × 10^−19^
DR93_RS01060	1.06133775	0.000104568	2.118819846	9.35 × 10^−18^	3.47595538	1.73 × 10^−34^
DR93_RS01080	2.863667052	1.89 × 10^−24^	3.23230808	6.80 × 10^−35^	1.040734492	2.79 × 10^−6^
DR93_RS01105	3.527734039	3.38 × 10^−34^	3.734578632	7.92 × 10^−43^	1.853393481	4.01 × 10^−14^
DR93_RS01265	2.31268298	3.19 × 10^−17^	3.078186912	1.02 × 10^−32^	1.226883989	9.29 × 10^−8^
DR93_RS01335	1.072518692	3.93 × 10^−5^	1.508544794	8.50 × 10^−12^	3.455316528	6.18 × 10^−36^
DR93_RS01345	1.179114647	5.95 × 10^−6^	2.286846072	2.63 × 10^−21^	2.680321649	1.14 × 10^−24^
DR93_RS01350	1.005942914	9.53 × 10^−5^	2.390158993	9.00 × 10^−23^	2.694262685	7.25 × 10^−25^
DR93_RS01550	2.216966779	6.03 × 10^−16^	1.842854473	1.57 × 10^−15^	1.567167873	7.61 × 10^−11^
DR93_RS01555	1.576489563	6.12 × 10^−9^	1.373914253	2.69 × 10^−10^	1.513915826	4.73 × 10^−10^
DR93_RS01890	1.853293199	4.33 × 10^−12^	2.561199115	4.71 × 10^−25^	2.401361167	6.54 × 10^−21^
DR93_RS02405	1.162875728	7.56 × 10^−6^	1.465590583	1.35 × 10^−11^	2.833206791	7.21 × 10^−27^
DR93_RS02410	1.266301067	1.33 × 10^−6^	1.931567298	9.10 × 10^−17^	2.81916466	1.33 × 10^−26^
DR93_RS02420	2.007607392	9.90 × 10^−14^	2.392099239	1.13 × 10^−22^	2.776896263	5.31 × 10^−26^
DR93_RS02425	2.003149255	1.17 × 10^−13^	3.010341713	1.80 × 10^−31^	2.730737645	2.47 × 10^−25^
DR93_RS02430	2.126106947	7.09 × 10^−15^	3.107608862	8.47 × 10^−33^	3.349434591	3.66 × 10^−34^
DR93_RS02435	2.056416582	3.41 × 10^−14^	2.924234663	3.48 × 10^−30^	3.364840925	1.48 × 10^−34^
DR93_RS02440	1.6016142	1.69 × 10^−9^	2.505759716	2.94 × 10^−24^	3.045822372	8.69 × 10^−30^
DR93_RS02810	1.773573597	3.68 × 10^−11^	1.855133127	1.14 × 10^−15^	2.114797579	4.16 × 10^−17^
DR93_RS03040	1.023633392	7.36 × 10^−5^	1.337102626	2.75 × 10^−10^	1.131848211	4.16 × 10^−7^
DR93_RS03195	2.261538459	1.03 × 10^−16^	3.223987332	8.64 × 10^−35^	1.709106196	1.34 × 10^−12^
DR93_RS03205	2.347601232	9.68 × 10^−18^	2.916439765	3.49 × 10^−30^	2.572537701	3.57 × 10^−23^
DR93_RS03675	1.337254132	4.20 × 10^−7^	1.001977031	3.72 × 10^−7^	1.701928337	2.39 × 10^−12^
DR93_RS03695	1.397031473	1.74 × 10^−7^	1.017698698	5.63 × 10^−7^	1.12935856	9.38 × 10^−7^
DR93_RS03700	1.472015537	7.48 × 10^−8^	1.109006422	1.45 × 10^−7^	1.44187039	4.87 × 10^−9^
DR93_RS04225	1.105527311	1.93 × 10^−5^	1.981916925	2.54 × 10^−17^	1.805467892	1.10 × 10^−13^
DR93_RS04555	2.739626381	1.06 × 10^−22^	4.517172961	8.20 × 10^−56^	3.36344786	1.61 × 10^−34^
DR93_RS04560	2.923929238	2.86 × 10^−25^	4.240364018	6.25 × 10^−51^	3.289409974	1.99 × 10^−33^
DR93_RS04565	2.763160453	3.82 × 10^−23^	3.639600609	3.38 × 10^−41^	4.040042399	2.07 × 10^−45^
DR93_RS04575	1.52667091	8.39 × 10^−9^	2.694698273	9.68 × 10^−27^	3.741770042	1.79 × 10^−40^
DR93_RS04580	1.600248728	1.78 × 10^−9^	2.398119676	2.42 × 10^−22^	2.763202417	1.14 × 10^−25^
DR93_RS04585	1.326543205	4.34 × 10^−7^	2.439108058	4.90 × 10^−23^	2.62459844	7.73 × 10^−24^
DR93_RS04775	1.14670014	1.23 × 10^−5^	1.119314906	3.49 × 10^−8^	1.991025432	1.44 × 10^−15^
DR93_RS05015	1.043165562	5.30 × 10^−5^	2.178629643	7.03 × 10^−20^	1.436144061	7.88 × 10^−10^
DR93_RS05020	1.283712871	8.98 × 10^−7^	2.416851449	5.41 × 10^−23^	1.735608723	6.84 × 10^−13^
DR93_RS05025	1.135726352	1.18 × 10^−5^	2.257833392	7.33 × 10^−21^	1.825776982	6.35 × 10^−14^
DR93_RS05150	1.804453136	1.82 × 10^−11^	3.009276252	2.23 × 10^−31^	3.103012813	1.28 × 10^−30^
DR93_RS05195	1.420050524	1.07 × 10^−7^	2.376038093	4.52 × 10^−22^	1.925846576	1.56 × 10^−14^
DR93_RS05705	1.298339374	7.18 × 10^−7^	1.282803532	8.22 × 10^−10^	1.538167553	9.57 × 10^−11^
DR93_RS06025	1.185357467	6.00 × 10^−6^	2.384507023	1.55 × 10^−22^	2.019440909	5.57 × 10^−16^
DR93_RS06195	1.408227525	1.01 × 10^−7^	2.751222313	1.65 × 10^−27^	2.064561415	1.45 × 10^−16^
DR93_RS06720	1.917560473	1.43 × 10^−12^	2.282361966	5.12 × 10^−21^	3.126199732	7.32 × 10^−31^
DR93_RS07490	2.610961283	9.93 × 10^−21^	2.729580743	6.28 × 10^−27^	3.65328795	2.59 × 10^−38^
DR93_RS07905	1.299563066	8.97 × 10^−7^	1.346558535	4.84 × 10^−10^	1.168936831	3.21 × 10^−7^
DR93_RS08095	1.771012993	4.60 × 10^−11^	2.666650872	2.04 × 10^−26^	3.21034127	4.17 × 10^−32^
DR93_RS08315	1.864766362	4.90 × 10^−12^	1.89146965	3.66 × 10^−16^	2.560782353	1.03 × 10^−22^
DR93_RS08770	2.105473353	1.32 × 10^−14^	1.977009627	5.01 × 10^−17^	2.849612859	1.19 × 10^−26^
DR93_RS08775	2.322898983	9.86 × 10^−16^	2.500148293	8.07 × 10^−22^	2.325257282	1.96 × 10^−17^
DR93_RS08780	2.888806297	8.61 × 10^−24^	3.313045233	8.16 × 10^−35^	2.819940541	4.33 × 10^−25^
DR93_RS09510	1.456867436	4.04 × 10^−8^	2.270730748	5.86 × 10^−21^	2.247823088	1.11 × 10^−18^
DR93_RS10225	1.527484206	7.69 × 10^−9^	2.667418562	1.86 × 10^−26^	2.339976968	4.56 × 10^−20^
DR93_RS10235	1.661578582	4.30 × 10^−10^	2.010403353	1.05 × 10^−17^	1.84311747	5.05 × 10^−14^

**Table 3:** DEGs that were downregulated in each infection stage compared with the control group.

Gene ID	min20_vs._min0		min30_vs._min0		min120_vs._min0	
	logFC	*p*-Value	logFC	*p*-Value	logFC	*p*-Value
DR93_RS00310	−1.22647431	9.04 × 10^−6^	−1.572250304	1.03 × 10^−5^	−1.784751618	1.07 × 10^−8^
DR93_RS00715	−1.282575153	3.39 × 10^−6^	−1.526449875	2.45 × 10^−5^	−1.358938206	2.77 × 10^−5^
DR93_RS00850	−2.779676581	4.14 × 10^−22^	−2.886496766	8.29 × 10^−19^	−2.530637518	2.78 × 10^−16^
DR93_RS00855	−2.61834377	5.10 × 10^−20^	−2.937338829	1.28 × 10^−19^	−2.558147849	1.32 × 10^−16^
DR93_RS00860	−2.247189319	1.72 × 10^−15^	−2.131052142	2.04 × 10^−10^	−2.015826525	9.26 × 10^−11^
DR93_RS00865	−2.409017507	2.26 × 10^−17^	−2.276979229	5.03 × 10^−12^	−2.317699978	7.41 × 10^−14^
DR93_RS00870	−1.953174477	2.66 × 10^−12^	−1.381735299	0.000196292	−1.347753538	3.27 × 10^−5^
DR93_RS00895	−1.080852274	7.96 × 10^−5^	−2.035264043	9.71 × 10^−10^	−2.199795137	5.22 × 10^−13^
DR93_RS01585	−1.25069892	1.31 × 10^−5^	−1.220059397	0.002730484	−1.809440337	6.55 × 10^−8^
DR93_RS02055	−1.255679843	5.79 × 10^−6^	−1.44637406	6.94 × 10^−5^	−1.156969158	0.000564594
DR93_RS02220	−1.987159654	8.04 × 10^−6^	−2.092577218	0.000160308	−2.293196376	0.000115102
DR93_RS02225	−1.578374314	2.25 × 10^−8^	−1.915517051	2.85 × 10^−8^	−1.832216506	1.27 × 10^−8^
DR93_RS02230	−1.282376477	4.26 × 10^−6^	−1.596918568	6.83 × 10^−6^	−1.49377855	4.09 × 10^−6^
DR93_RS02355	−1.79904637	2.63 × 10^−10^	−1.580792263	1.50 × 10^−5^	−1.323103384	7.91 × 10^−5^
DR93_RS02925	−1.002597105	0.000312063	−1.306055801	0.000461551	−1.123445908	0.001031075
DR93_RS03000	−1.324974955	1.65 × 10^−6^	−1.487134988	4.36 × 10^−5^	−1.49735502	2.82 × 10^−6^
DR93_RS03135	−1.511757049	4.51 × 10^−8^	−1.267310844	0.000772233	−1.783378696	1.08 × 10^−8^
DR93_RS03145	−1.317110278	1.94 × 10^−6^	−1.464588214	5.28 × 10^−5^	−1.33520997	3.97 × 10^−5^
DR93_RS03150	−1.475875314	1.02 × 10^−7^	−1.518610036	2.42 × 10^−5^	−1.40393369	1.41 × 10^−5^
DR93_RS03535	−2.769956856	4.93 × 10^−22^	−3.511717223	1.76 × 10^−27^	−1.428513597	9.70 × 10^−6^
DR93_RS03540	−2.421974255	1.01 × 10^−17^	−3.289082981	1.18 × 10^−24^	−1.414427548	1.12 × 10^−5^
DR93_RS03545	−2.049769548	2.18 × 10^−13^	−2.941036907	5.09 × 10^−20^	−1.399540572	1.40 × 10^−5^
DR93_RS04175	−1.11974413	5.13 × 10^−5^	−1.184348018	0.002648883	−1.365642259	2.30 × 10^−5^
DR93_RS04610	−1.255351867	5.41 × 10^−6^	−1.531889936	2.28 × 10^−5^	−1.466924464	4.41 × 10^−6^
DR93_RS04615	−1.337046708	1.36 × 10^−6^	−1.617303153	5.18 × 10^−6^	−1.665613403	1.25 × 10^−7^
DR93_RS04760	−1.031841995	0.000175223	−1.749999118	2.68 × 10^−7^	−2.227992415	3.59 × 10^−13^
DR93_RS05300	−1.564303898	1.69 × 10^−8^	−1.889269946	2.69 × 10^−8^	−1.551383853	1.04 × 10^−6^
DR93_RS05305	−1.764214686	2.24 × 10^−10^	−2.208939023	2.19 × 10^−11^	−1.508058464	2.34 × 10^−6^
DR93_RS05310	−2.2552225	1.02 × 10^−15^	−2.624427397	4.69 × 10^−16^	−1.631924843	2.19 × 10^−7^
DR93_RS05315	−2.174299836	1.07 × 10^−14^	−2.268440114	7.74 × 10^−12^	−1.116242359	0.000946037
DR93_RS05320	−2.272560324	7.14 × 10^−16^	−2.710755424	6.12 × 10^−17^	−2.120989732	6.30 × 10^−12^
DR93_RS05325	−1.921040036	5.26 × 10^−12^	−2.135737075	1.18 × 10^−10^	−1.659189028	1.23 × 10^−7^
DR93_RS05520	−1.034115931	0.000166235	−1.960117081	3.12 × 10^−9^	−2.629448477	6.55 × 10^−18^
DR93_RS05755	−1.072850033	0.000106606	−1.538579127	1.67 × 10^−5^	−1.288190385	8.36 × 10^−5^
DR93_RS05760	−1.086467496	8.40 × 10^−5^	−1.552688867	1.30 × 10^−5^	−1.345585343	3.13 × 10^−5^
DR93_RS06055	−1.642343017	3.21 × 10^−9^	−1.405942492	0.000124317	−1.329378091	4.38 × 10^−5^
DR93_RS06440	−2.274992575	6.38 × 10^−16^	−1.643051948	3.34 × 10^−6^	−1.020130843	0.003135403
DR93_RS06660	−1.384837071	2.22 × 10^−6^	−1.430183454	0.000258917	−1.449409771	3.69 × 10^−5^
DR93_RS06690	−1.445901523	8.68 × 10^−7^	−1.604658799	2.74 × 10^−5^	−1.367629593	0.000131911
DR93_RS06695	−1.630267283	1.01 × 10^−8^	−1.950673646	2.41 × 10^−8^	−2.52360238	1.23 × 10^−14^
DR93_RS06745	−1.377069537	7.48 × 10^−7^	−1.48805423	4.45 × 10^−5^	−2.404082681	4.18 × 10^−14^
DR93_RS07090	−1.124825417	5.48 × 10^−5^	−1.208987156	0.001912183	−1.361415971	3.19 × 10^−5^
DR93_RS07180	−2.051307734	3.13 × 10^−13^	−2.413672296	2.35 × 10^−13^	−1.946253364	5.58 × 10^−10^
DR93_RS07185	−1.452692929	1.62 × 10^−7^	−1.329258816	0.000413249	−1.082770175	0.001539617
DR93_RS07425	−1.090886247	7.82 × 10^−5^	−1.306997728	0.00058163	−1.719149213	3.95 × 10^−8^
DR93_RS07675	−1.748506347	3.52 × 10^−10^	−1.699187114	1.13 × 10^−6^	−2.015466981	8.82 × 10^−11^
DR93_RS07715	−1.396311848	3.94 × 10^−7^	−1.691827493	1.23 × 10^−6^	−1.662953886	1.15 × 10^−7^
DR93_RS08070	−3.353697746	1.75 × 10^−30^	−4.187865095	4.87 × 10^−38^	−3.252693259	4.03 × 10^−26^
DR93_RS08075	−3.158653222	1.39 × 10^−27^	−3.477932516	5.16 × 10^−27^	−2.302650666	6.14 × 10^−14^
DR93_RS08185	−1.087832652	8.10 × 10^−5^	−1.717575245	6.11 × 10^−7^	−1.677397302	8.47 × 10^−8^
DR93_RS08245	−1.085127686	8.48 × 10^−5^	−1.327065442	0.000395768	−1.633453757	1.93 × 10^−7^
DR93_RS08250	−1.590190722	8.76 × 10^−8^	−1.827852132	8.75 × 10^−7^	−2.376032448	2.60 × 10^−11^
DR93_RS08405	−1.416932957	3.10 × 10^−7^	−1.547935451	1.58 × 10^−5^	−1.200901072	0.000311803
DR93_RS09055	−1.408615657	7.72 × 10^−7^	−1.66436419	4.21 × 10^−6^	−1.303918805	0.000127531
DR93_RS09515	−1.116768397	5.03 × 10^−5^	−1.679582198	1.30 × 10^−6^	−1.564442053	7.09 × 10^−7^
DR93_RS09525	−1.59281361	1.10 × 10^−8^	−1.436246155	8.84 × 10^−5^	−1.014227896	0.003561264
DR93_RS09530	−1.823516793	7.56 × 10^−11^	−1.933344071	1.16 × 10^−8^	−1.193393201	0.000371375
DR93_RS10435	−2.015964956	5.14 × 10^−13^	−2.644345525	2.05 × 10^−16^	−2.8714223	6.73 × 10^−21^

## Data Availability

The raw strand-specific RNA-seq reads generated in this study have been deposited in the NCBI Sequence Read Archive (SRA) under BioProject accession number PRJNA1493802. The 12 RNA-seq libraries are associated with BioSample accession numbers SAMN61575621–SAMN61575632 and SRA run accession numbers SRR39578895–SRR39578906. All other data supporting the findings of this study are included in the article and its Supplementary Materials.

## Supplementary Materials

The following supporting information can be downloaded at https://www.mdpi.com/article/10.3390/microorganisms14071582/s1, Table S1: Statistical table of quality control data; Table S2: Statistical table of comparison results; Table S3: Differentially expressed genes (DEGs) at each infection stage compared with the control group.
